# The KATP channel opener, nicorandil, ameliorates brain damage by modulating synaptogenesis after ischemic stroke

**DOI:** 10.1371/journal.pone.0246019

**Published:** 2021-01-26

**Authors:** Yuanzheng Zhao, Zhuoying Yang, Yuanhong He, Ruonan Sun, Heping Yuan

**Affiliations:** Department of Neurology, The Fifth Affiliated Hospital of Zhengzhou University, Zhengzhou, Henan, China; Ehime University Graduate School of Medicine, JAPAN

## Abstract

With population growth and aging, more and more patients with cerebral infarction have varying degrees of disability. ATP-sensitive potassium (KATP) channels regulate many cellular functions by coupling metabolic status with cell membrane electrical activity. Nicorandil (N-(2-hydroxyethyl)-nicotinamide nitrate) is the first KATP channel opener approved for clinical use. It has been reported that it might exert protective effects on the cerebral infarction by increasing cerebral blood flow and reducing inflammation. However, only a few studies explored its role in synaptogenesis. We made the rat model of middle cerebral artery occlusion (MCAO). Nicorandil was administered to rats via oral administration immediately after the surgery at a dose of 7.5 mg/kg and then daily for the next days. Infarct volume, cerebral edema, neurological deficits, cognitive impairment, and the level of Synaptophysin (SYP)、Growth associated protein-43 (GAP43) and neuronal nuclear antigen (NeuN) levels were measured to evaluate the effect of nicorandil. Our data showed that nicorandil treatment could decrease brain damage, improve learning and memory, and increase SYP、GAP43 and NeuN level. Taken together, we propose that nicorandil, as an opener of the KATP channel, provides a neuroprotective role in MCAO by promoting synaptic connections.

## Introduction

Ischemic stroke is one of the leading causes of death worldwide and is characterized by high morbidity, mortality and disability rates. It places a heavy burden and suffering on society and family [1]. However, the main treatment is still ultra-early stage thrombolytic therapy. Only a small number of patients are treated. Effective therapeutic options for the clinical management of ischemic stroke remain limited [2].

KATP channels are widely distributed in various tissues, including neurons, skeletal muscle, smooth muscle and pancreatic islet beta cells. In the central nervous system, KATP channels are widely distributed in different brain regions, including the substantia nigra, neocortex, hippocampus and hypothalamus [3, 4]. Activation of KATP channels hyperpolarizes neurons, prevents excitotoxicity, stabilizes the membrane potential and reduces the ionic imbalance. It also prevents neuronal damage and neurodegeneration caused by anoxic membrane depolarization and excitotoxicity [5, 6]. Nicorandil is a mitoKATP channel opener with nitrate-like properties and a proven anti-angina agent. Previous studies have shown that neuronal cells express 6- to 7-fold more mitoKATPs as the myocardium [7]. Some studies have demonstrated that nicorandil increases cerebral blood flow, and prevents neural death through anti-inflammatory, reducing calcium overload and stabilizing the BBB, thereby generating neuroprotective effects in cerebral ischemia [8–10].

Ischemic stroke can cause a series of dysfunction such as speech, movement, learning and so on. The compensatory mechanism appears in the body, which makes the function recover, especially the role of synaptic plasticity [11]. When the axon is destroyed, the protein level related to synaptic growth and reconstruction will change in the body. Through the interaction between proteins, the information transmission is completed, and the synaptic plasticity is improved. Among them, SYP and GAP43 are closely related to synaptic transmission [12]. In our study, we investigated whether nicorandil could promote synaptogenesis in a rat middle cerebral artery occlusion (MCAO) model.

## Materials and methods

### Animals

Male SD (Sprague-Dawley) rats (6–8 weeks old, 200–240 g) were used, and they were purchased from the Animal Experimental Center of Zhengzhou University. They were housed in clear cages (5 per cage) and received a standard diet and water. The environmental temperature was controlled at 20–25°C with a 12 h light and 12 h dark cycle. All animal procedures were carried out according to protocols approved by the Animal Care and Use Committee of Zhengzhou University (approval No.: SYXK2005-0012). All efforts were made to minimize the number of animals used and their suffering.

### Transient MCAO model and treatment

Rats were subjected to a 90-min MCAO procedure using the intraluminal suture model [13]. Briefly, we anesthetized rats with 1.5% isoflurane in a 70% nitrous oxide and 30% oxygen mixture using a face mask. Then, we made a ventral midline cervical incision, exposed the right common carotid artery (CCA), separated the vagus nerves, and made a small incision in the internal carotid artery. Then, the origin of the middle cerebral artery (MCA) was occluded by inserting a 4–0 nylon monofilament. We withdrew the monofilament after 90 min. Laser Doppler flowmetry (Moor Instruments, Devon, UK) was used to measure the successful establishment of the MCAO model. An incision was made in the scalp to expose the skull. It was fixed to the surface of the skull on the left side (2 mm posterior, 4 mm lateral to bregma) and the criterion was an approximately 80% decrease in cerebral blood flow. Rats in the Sham group underwent the same surgical procedure, but the filament was inserted into the initial segment of the middle cerebral artery and immediately withdrawn. Body temperature was kept at 37 ± 0.5°C using a controlled heating plate during experiments. The rats were divided into Sham group (n = 56), MCAO group (n = 56), MCAO+Nicorandil group (n = 56), and MCAO+Vehicle group (n = 56) according to the randomization principle. Nicorandil was dissolved in 0.9% saline, and it was administered to rats via oral administration immediately after the surgery at a dose of 7.5 mg/kg and then daily for the next days before being sacrificed [14], while the MCAO+Vehicle group received the same volume of saline only. 5-Bromo-2’-deoxyuridine (BrdU, 50 mg/kg, Sigma-Aldrich, St. Louis, MO, USA) was administered through an intraperitoneal injection 24 h after surgery and for 13 consecutive days [15].

### Evaluation of infarct volume

2,3,5-Triphenyltetrazolium chloride (TTC, Sigma-Aldrich, St. Louis, MO, USA) was dissolved in PBS at a concentration of 2%. After the rats were deeply anesthetized, the brains were removed rapidly and cut into five coronal slices with a thickness of 2 mm using a rodent brain matrix. Brain slices were immersed in TTC at 37°C for 30 min, fixed with 4% paraformaldehyde for 24 h, and then photographed with a digital camera. The red area was the normal area, and the white area was the cerebral infarction area. The infarct volume was calculated by using ImageJ software (NIH, Bethesda, MD, USA), and the infarct volume percentage was computed as total infarct volume/contralateral hemisphere volume×100% [16].

### Assessment of brain water content

We sacrificed six rats from each group 72 h after MCAO to measure the brain water content. The brain was rapidly removed, and the right hemisphere was then isolated from the brain. It was immediately weighed to obtain the wet weight. After 24 h of drying in an oven at 100°C, the hemisphere was weighed again to obtain the dry weight. We calculated the brain water content with the formula: (wet weight-dry weight)/wet weight×100% [17].

### Assessment of neurological function

The modified neurological severity score (mNSS) was calculated to assess the neurological function as described in a previous study [18]. The test was performed 1, 3, 7, and 14 days after MCAO by a recorder blinded to the treatments and experimental groups. The score combines the results of sensory, motor, reflex and balance tests. The total score ranged from 0–18 points, with a higher score indicating a more severe injury.

### Morris Water Maze (MWM)

The MWM task was performed from day 15 to day 20 after MCAO [19]. It was performed in a circular pool (diameter of 150 cm and height of 50 cm) filled with water (20–22°C, 30 cm in depth) and divided into four equal quadrants (quadrants I, II, III, and IV). The escape platform (12 cm in diameter) was located 2 cm below the water surface in the middle of quadrant I. In the first five days, each rat was randomly placed in one of the four quadrants and allowed to swim for a maximum of 120 s to find the platform. If the rat did not find the platform, it would be guided to the platform and allowed to stay on it for 10 s; in this case, the escape latency was recorded as 120 s [20]. The swimming speed and escape latency to find the platform was recorded. The probe trial was performed 24 h after the last hidden platform test. On day 20, the platform was removed, and rats were subjected to one 120 s retention probe trial. The time each rat spent in quadrant I was recorded and quantified. The investigator was blinded to the experimental conditions.

### Immunofluorescence staining

After deep anesthetized, rats were perfused transcardially with saline and 4% paraformaldehyde. Then, brains were dissected out and post-fixed with 4% paraformaldehyde 24 h, and dehydrated in 30% sucrose solution respectively until the tissues sank to the bottom. After embedding with OCT, they were cut into 20-μm-thick floating sections every 480 μm by cryoultramicrotomy (CM1100, Leica Biosystems, Germany). Tissue sections were stored in antifreeze buffer at -20°C for later use. Brain sections were incubated with PBST (0.1% Triton X-100 in 0.01 M PBS) and then blocked with 1% bovine serum albumin for 30 min. For BrdU immunofluorescence staining, sections were placed in 2 N HCL (37°C, 30 min) and washed with borate saline buffer (pH 8.4) twice for 5 min each. Sections were incubated with a mouse anti-BrdU (1:1000, Cell Signaling Technology), a rabbit anti-SYP (1:150, Abcam), rabbit anti-NeuN (1:300, Abcam), or rabbit anti-GAP43 (1:500, Abcam) antibody overnight at 4°C. After rinses with PBS, sections were incubated with donkey anti-mouse IgG H&L (Alexa Fluor 594; 1:500, Abcam) or goat anti-rabbit IgG H&L (Alexa Fluor 488; 1:500, Abcam) for 1 h at room temperature. Then, sections were washed with PBS and mounted on coverslips with a drop of mounting medium that contained 1.5 μg/mL 4’,6-diamidino-2-phenylindole (DAPI; Santa Cruz). For each section, we randomly chose three nonoverlapping 20× fields to measure the BrdU positive area, the SYP positive area, and the GAP43 positive area. The BrdU positive area was measured in the region of the SVZ (subventricular zone). The NeuN positive area was measured in the peri-infarct area. The SYP and GAP43 fluorescence intensities were measured in the hippocampal CA1 region. An investigator blinded to the experimental treatments measured stained sections with a fluorescence microscope (ZEISS Scope A1, ZEISS, Germany).

### RT-PCR

Brains were quickly dissected and specified brain regions in the ipsilesional brain hemisphere, including the peri-infarct zone and hippocampus, were separated and collected. Total RNA was extracted using a Trizol kit according to the manufacturer’s recommendations and then reverse transcribed to cDNAs in 20 μl reaction volumes. The cDNA templates were subjected to PCR amplification with specific primers for rat NeuN, SYP and GAP43 and a premixed PCR kit. The following specific primers were used (Sangon Biotech, Shanghai, China):

NeuN, (f) 5’-CCC TCC CTC AGC AGA CAC -3’, (r) 5’-TCA GCA GCC GCA TAG ACT CTA CC -3’;

SYP, (f) 5’- GCT GTG TTT GCC TTC CTC TAC TCC-3’, (r) 5’- GCC TGT CTC CTT GAA CAC GAA CC-3’;

GAP43, (f) 5’-TGT GGA GAA GAA GGA GGG AGA TGG-3’, (r) 5’-AGG ACG GCG AGT TAT CAG TGG TAG -3’;

GAPDH, (f) 5’- GAC ATG CCG GGA GAA AC-3’, (r) 5’-AGC CCA GGA TAC CCT TTA GT -3’.

The cycling parameters were denaturation at 95°C for 30 s followed by 35 cycles of incubations at 95°C for 30 s, 61°C for 30 s and 75°C for 1 min. The final extension step was performed at 72°C for 10 min, and each cDNA sample was analyzed in triplicate. PCR products were separated on 2% agarose gels, and GAPDH was amplified under similar conditions. Gels were analyzed using an Alpha Innotech imager (BIO-RAD) [21].

### Western blot

Specified brain regions in the ipsilesional brain hemisphere, including the peri-infarct area and hippocampus, were freshly dissected in ice-cold PBS and lysed in RIPA lysis buffer. The homogenates were centrifuged (14000×g, 10 min, 4°C), and proteins were collected from the supernatant. Protein samples from each group were loaded onto SDS-PAGE gels (4%-12%), separated electrophoretically and transferred onto polyvinylidene difluoride (PVDF) membranes. The membranes were blocked with 5% nonfat milk for 1 h at room temperature and incubated overnight at 4°C with one of the following primary antibodies: rabbit anti-NeuN (1:5000, Abcam), rabbit anti-SYP (1:20000, Abcam), rabbit anti-GAP43 (1:1000, Abcam), or rabbit anti-GAPDH (1:2000, Sangon Biotech, Shanghai, China). After washes with Tris-buffered saline containing 0.1% Tween 20 (TBST), membranes were incubated with secondary antibodies conjugated with horseradish peroxidase for 1 h at room temperature and then washed again. Protein bands were detected with an enhanced chemiluminescence (ECL) kit (CWBIO, Beijing, China). An investigator blinded to the experimental groups quantified the optical density after normalization to GAPDH by using Gel Analysis V 2.02 software 9 (Clinx Science Instruments, Shanghai, China).

### Statistical analysis

All analyses were performed with SPSS software version 13.0. All data were tested for normality using the Kolmogorov-Smirnov test. All data are reported as means ± SD. We used one-way ANOVA followed by the least significant difference (LSD) test to analyze changes in the infarct volume, brain water content, time spent in the target zone, RT-PCR and western blot data. We used repeated measures ANOVA followed by the LSD test to analyze the mNSS, escape latency, and swimming speed among different groups. A p-value<0.05 was considered statistically significant.

## Results

### Nicorandil treatment decreases brain damage in rats after MCAO

**Fig 1:**
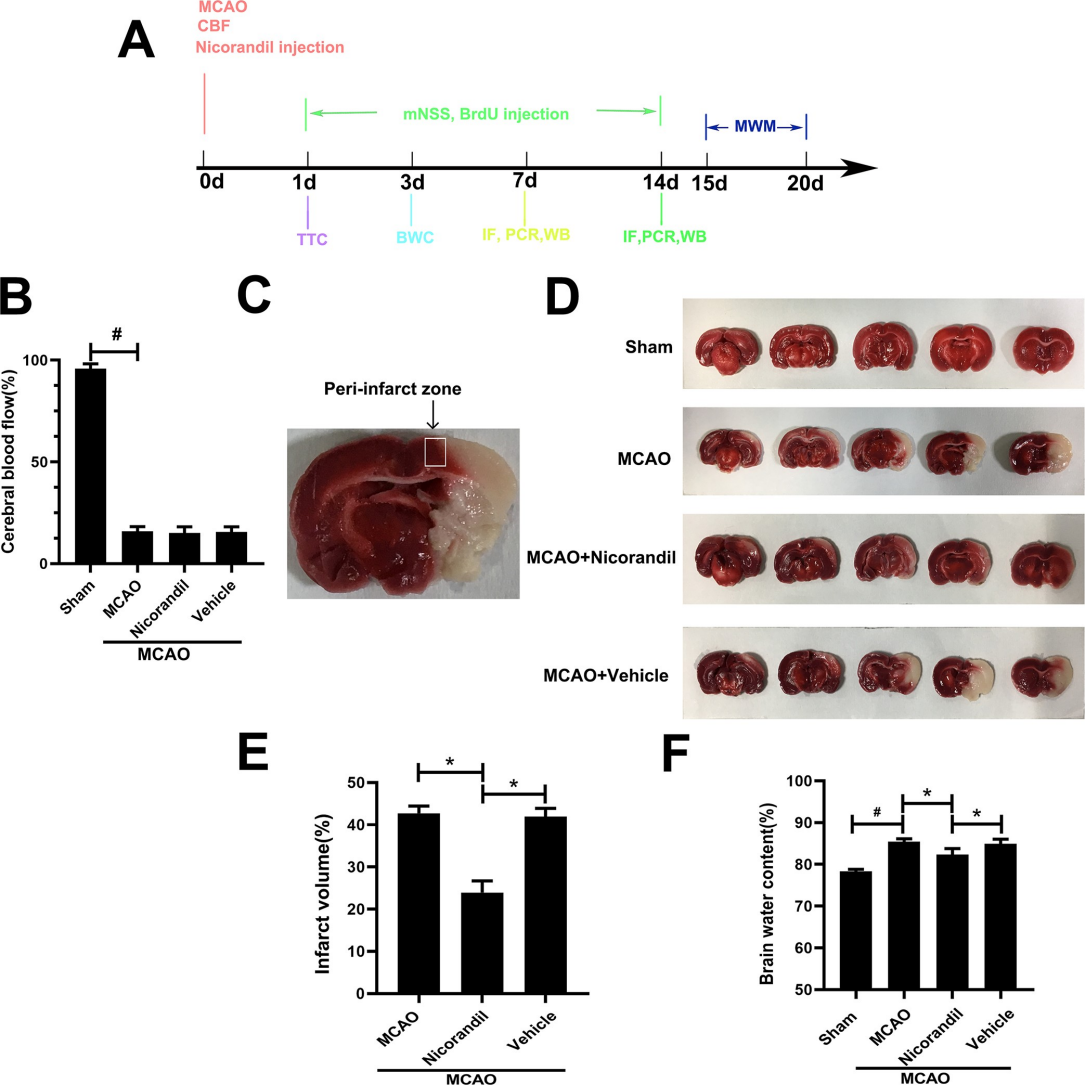
The nicorandil treatment alters the infarct volume and brain water content after MCAO. (A) Schematic representation of experimental design. (B) Changes of cerebral blood flow (CBF) of all groups. #p<0.05 compared with the Sham group, n = 56/group. (C) Representative image of the peri-infarct zone. (D) Representative TTC staining in each group at 24 h after MCAO. (E) Analysis of the infarct volume of all groups, *p<0.05, n = 6 rats per group. (F) Quantification of the brain water content. Data are presented as means ± SD. *p<0.05 compared with the MCAO group and MCAO+Vehicle group; #p<0.05 compared with the Sham group, n = 6 rats per group.

The mortality of each group was 4/56 (7.1%) in the Sham group, 12/56 (21.4%) in the MCAO group, 11/56 (19.6%) in the MCAO+Nicorandil group and 13/56 (23.2%) in the MCAO+Vehicle group. The cerebral blood flow decreased to about 20% of the baseline level before the MCAO operation (Fig 1B). TTC staining revealed a significantly smaller infarct volume in the MCAO+Nicorandil group than in the other two MCAO-operated groups (Fig 1D and 1E). Rats that received nicorandil had lower brain water content than rats in the MCAO group and MCAO+Vehicle group (Fig 1F). A similar trend was observed in the MCAO group and MCAO+Vehicle group (Fig 1E and 1F).

### Administration of nicorandil reduces neurological deficits and improves learning and memory disorders after MCAO

**Fig 2:**
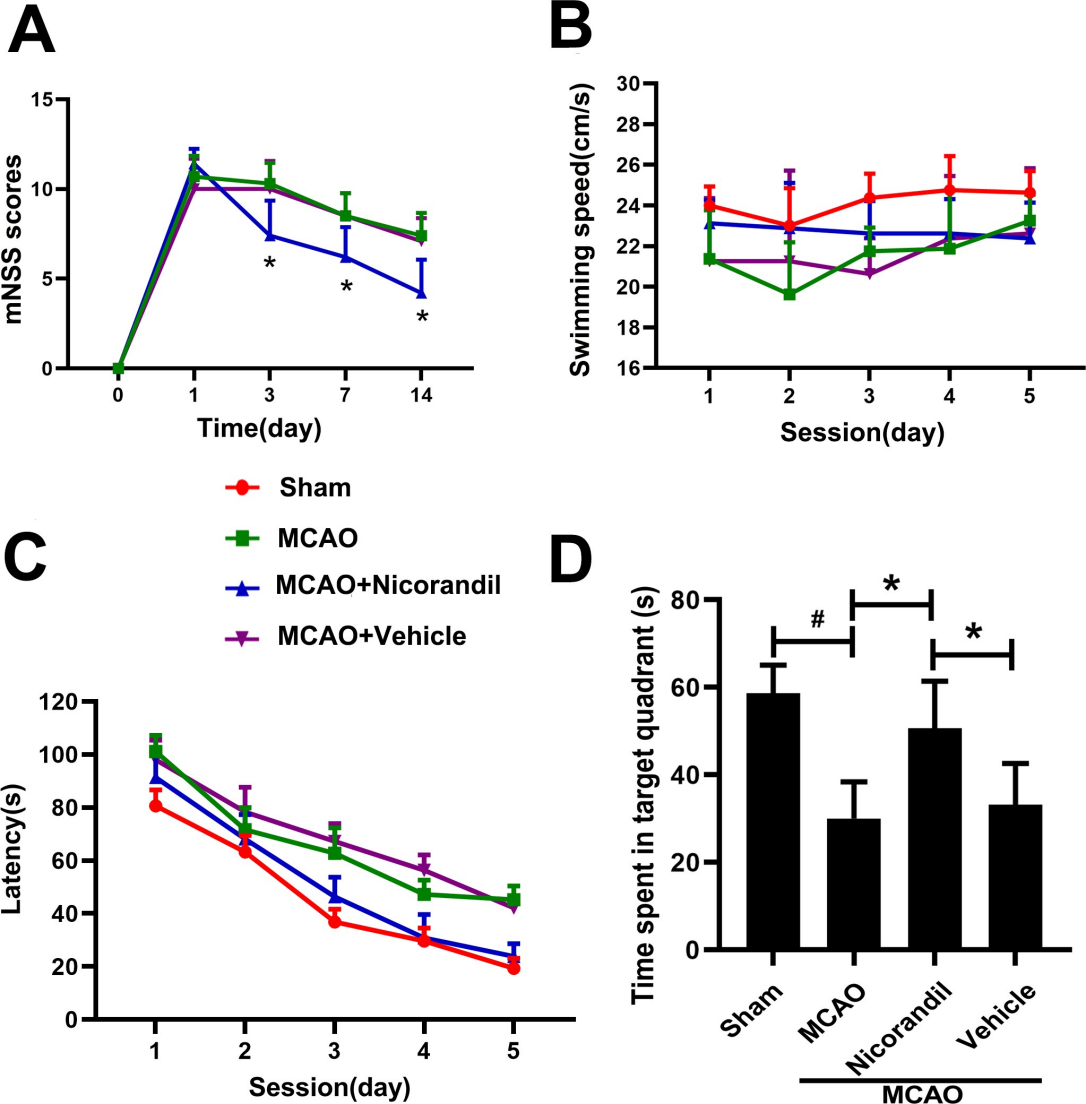
Effects of nicorandil on the mNSS test and MWM test. (A) Significantly lower mNSS scores were recorded for the nicorandil-treated MCAO rats than for the MCAO and saline-treated MCAO rats on days 3, 7, and 14 (*p<0.05, n = 10 rats per group). (B) Swimming speed of each group (p>0.05). (C) Escape latency of each group. (D) Time spent in the target quadrant (*p<0.05 compared with the MCAO group and MCAO+Vehicle group; #p<0.05 compared with the Sham group, n = 8 rats per group). Data are presented as means ± SD.

The nicorandil treatment improved neurological performance in mNSS tests compared with the MCAO group and MCAO+Vehicle group (Fig 2A). The average swimming speed of rats in each group was similar (Fig 2B), ensuring the fairness and validity of the MWM measurement. Compared to the Sham group, the escape latency of MCAO group was significantly increased. The nicorandil-treated rats displayed a significantly decreased escape latency to find the platform, and the extent of the decrease was more pronounced than in rats from the other two MCAO groups (Fig 2C). Besides, rats in the MCAO+Nicorandil group spent more time in the target quadrant. A significant difference was not observed between the MCAO group and MCAO+Vehicle group (Fig 2D).

### Nicorandil treatment increases NeuN expression in the peri-infarct area

**Fig 3:**
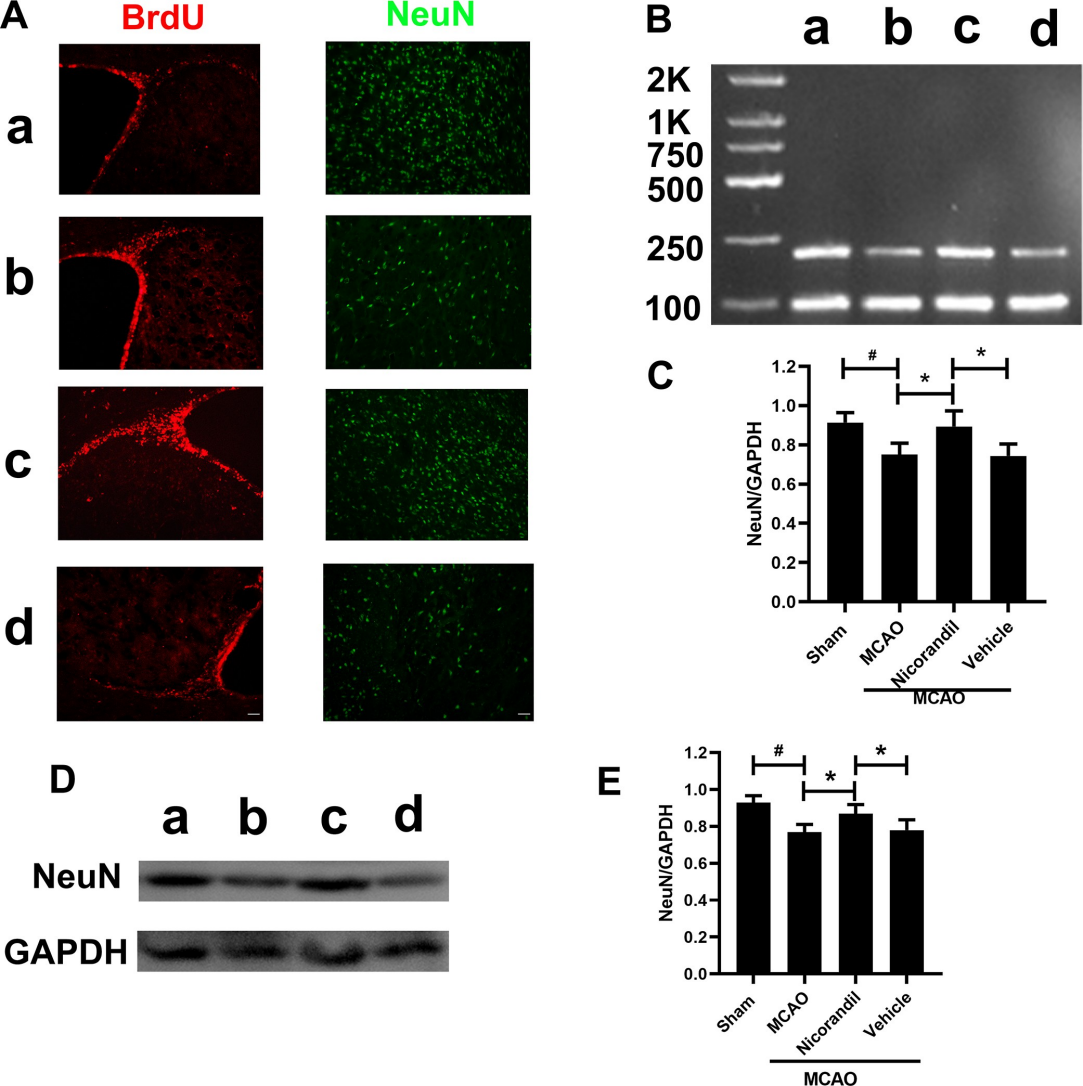
Nicorandil increase NeuN after MCAO. (A) Representative images of BrdU and NeuN on day 14 after MCAO. Scale bar = 100 μm. n = 6 rats per group. (B) Representative RT-PCR analysis of NeuN levels. The sizes of the NeuN and GAPDH products were 214 bp and 89 bp, respectively. (C) Analysis of the optical density (OD) of all groups. n = 6 rats per group. (D) Representative western blot showing NeuN levels. (E) Quantification of the western blot analysis. *p<0.05 compared with the MCAO group and MCAO+Vehicle group; #p<0.05 compared with the Sham group. n = 6 rats per group. Data are presented as means ± SD. a: Sham group; b: MCAO group; c: MCAO+Nicorandil group; d: MCAO+Vehicle group.

We analyzed NeuN levels using immunofluorescence staining, RT-PCR and western blot on day 14 after MCAO. Fig 3A presents the results of immunoreaction for BrdU and NeuN. RT-PCR results revealed lower levels of the NeuN mRNA in the MCAO group than in the Sham group. NeuN was expressed at higher levels in the MCAO+Nicorandil group than in the MCAO group and MCAO+Vehicle group. Fig 3D and 3E present western blot results. The quantification of band densities revealed lower levels of the NeuN protein in the MCAO group than in the Sham group. NeuN was expressed at higher levels in the nicorandil-treated rats than in those from the MCAO group and MCAO+Vehicle group. A significant difference in the levels of the NeuN protein was not observed between the MCAO group and MCAO+Vehicle group.

### SYP expression in rats after MCAO

**Fig 4:**
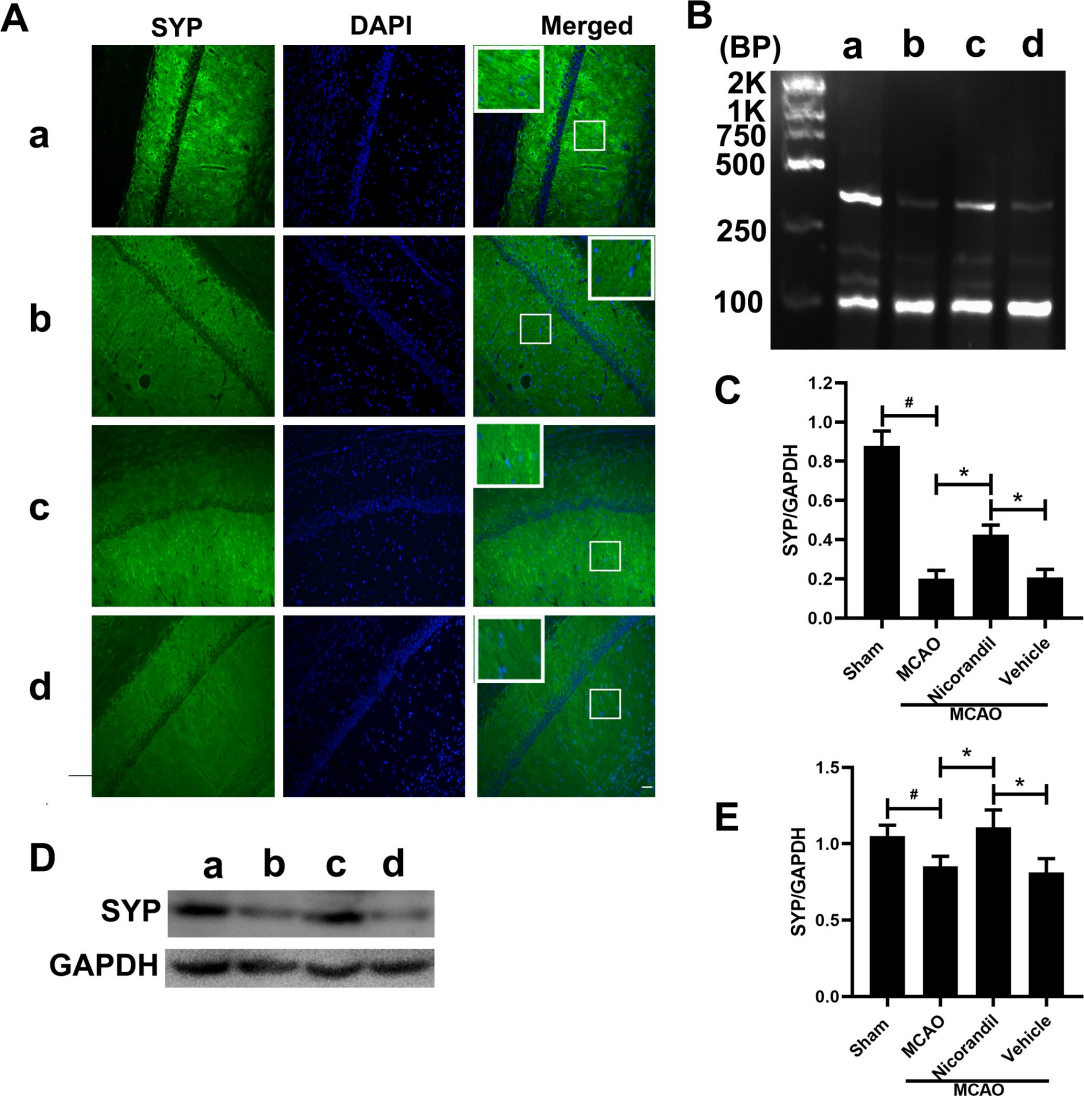
Nicorandil inhibits the decrease of SYP. (A) Representative photomicrographs of immunofluorescence staining for SYP in the hippocampal CA1 area on day 14 after MCAO. Scale bar = 100 μm. n = 6 rats per group. (B) Representative RT-PCR assay of SYP levels. The sizes of the SYP and GAPDH products were 351 bp and 89 bp, respectively. (C) Quantification of the optical density (OD) of all groups. *p<0.05 compared with the MCAO and MCAO+Vehicle groups; #p<0.05 compared with the Sham group. n = 6 rats per group. (D) Representative western blot showing SYP levels. (E) Quantification of the western blot analysis. *p<0.05 compared with the MCAO and MCAO+Vehicle groups; #p<0.05 compared with the Sham group. n = 6 rats per group. a: Sham group; b: MCAO group; c: MCAO+Nicorandil group; d: MCAO+Vehicle group. n = 6 rats per group. All values are presented as means ± SD.

We analyzed SYP levels using immunofluorescence staining, RT-PCR and western blot on day 14 after MCAO. Fig 4A presents the results of an immunoreaction for SYP in the hippocampal CA1 region. The nicorandil treatment increased SYP immunoreactivity. The RT-PCR analysis of the SYP mRNA revealed decreased SYP mRNA levels in the three MCAO-operated groups compared with the Sham group. Meanwhile, a significant difference was not observed between the MCAO group and MCAO+Vehicle group (Fig 4B and 4C). Fig 4D and 4E show the effects of nicorandil on the expression of SYP at the protein level. Higher SYP protein levels were detected in the Sham group, and ischemic stroke significantly decreased SYP levels. According to the statistical analysis, the nicorandil treatment prevented the decrease in SYP levels caused by ischemic stroke, as this group presented higher SYP levels than the other two MCAO-operated groups. However, the SYP level was still lower in the MCAO+Nicorandil group than in the Sham group.

### Changes in GAP43 expression in the hippocampus after MCAO

**Fig 5:**
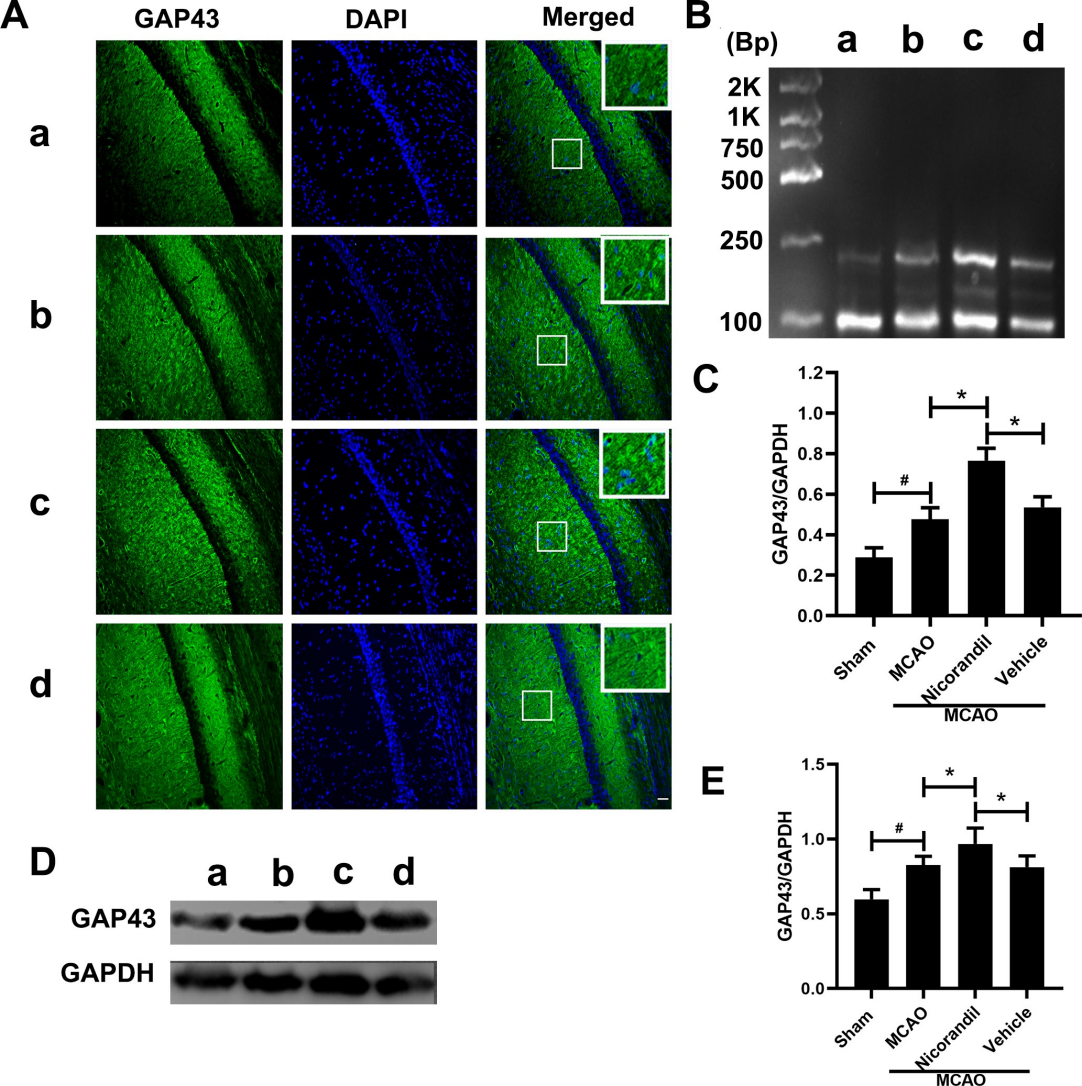
Nicorandil improves the level of GAP43 after MCAO. (A) Representative images show the immunofluorescence staining for GAP43. Scale bar = 100 μm. n = 6 rats per group. (B) Representative RT-PCR assay of GAP43 levels. The sizes of the GAP43 and GAPDH products were 203 bp and 89 bp, respectively. (C) Quantification of the RT-PCR data revealed the differences between all groups. *p<0.05 compared with the MCAO and MCAO+Vehicle groups; #p<0.05 compared with the Sham group. n = 6 rats per group. (D) Representative western blot showing GAP43 levels. (E) Quantification of the western blot analysis. *p<0.05 compared with the MCAO and MCAO+Vehicle groups; #p<0.05 compared with the Sham group. n = 6 rats per group. a: Sham group; b: MCAO group; c: MCAO+Nicorandil group; d: MCAO+Vehicle group.

In addition to detecting GAP43 immunofluorescence staining’s intensity, we analyzed GAP43 expression at the mRNA and protein levels on day 7 after MCAO. Fig 5A presents GAP43 immunofluorescence staining in the hippocampal CA1 area on day 7 after MCAO. Higher GAP43 immunoreactivity was observed in the MCAO group than in the Sham group. Meanwhile, the MCAO+Nicorandil group showed increased GAP43 immunoreactivity compared with the other MCAO-operated groups. The results of the RT-PCR assay of the GAP43 mRNA are shown in Fig 5B and 5C. Higher levels of the GAP43 mRNA were detected in the MCAO group than in the Sham group. Nicorandil induced a significant increase in GAP43 expression, and a significant difference was not observed between the MCAO group and MCAO+Vehicle group. Fig 5D and 5E illustrate the western blot results. The quantification of band densities revealed a higher level of the GAP43 protein in the MCAO group than in the Sham group. GAP43 was expressed at significantly higher levels in the MCAO+Nicorandil group than in the MCAO group and MCAO+Vehicle group. All values are presented as means ± SD.

## Discussion

In this study, nicorandil decreased brain damage, improved behavioural deficits and alleviated memory impairment after MCAO. Furthermore, nicorandil increased the expression of GAP43, and blocked the decrease in SYP levels. We also excluded the placebo effect throughout the experiment. Based on the results of our study, nicorandil is a potential therapeutic strategy for ischemic stroke.

The KATP channel is a type of potassium ion channel inhibited by ATP and activated by ADP, coupling the cell’s metabolic state (ATP/ADP ratio) with the electrical activity of the cell membrane [3]. In a stroke, a pathological state of hypoxia or inadequate nutrient delivery occurs, leading to a decrease in ATP levels and the activation of KATP channels. As early as 1993, some scholars proposed that the activation of KATP channel can regulate the cell membrane potential during hypoxia or ischemia, which helps to inhibit neurons’ pathological excitation and protect neurons from degeneration [5]. In recent years, the role of KATP channels in stroke has been mentioned successively [22, 23].

Our data showed that the infarct volume in the MCAO group is 42.74%, and the infarct volume in the nicorandil group is 20.77%. Gupta et al. showed that in vascular dementia model, oral administration of two doses of nicorandil (2 and 4mg/kg) showed a reduction in infarct volume, with a more significant reduction of 4mg/kg [24]. Kong et al. showed that nicorandil increased cell viability and reduced apoptosis rate in a concentration-dependent manner [25]. Li et al. also demonstrated that, in hypercholesterolemic rats, pharmacological preconditioning and postconditioning with nicorandil dose-dependently decreased I/R-induced myocardial necrosis and apoptosis [26]. We chose the oral nicorandil dose of 7.5 mg/kg, and showed a significant decrease in infarct volume compared with MCAO group. First, we have given enough doses. Second, the way of drug administration was oral, which could increase the absorption of nicorandil.

Meanwhile, we used mNSS to detect neurological deficits in rats, and the results showed that there was little change on the first day, and the neurological deficits were significantly improved on the 3rd, 7th, and 14th days. The mNSS is one of the most commonly used comprehensive scales in MCAO rat models. There are no other similar studies can be compared. Previous studies have shown that Kir6.1 knockdown aggravates cerebral I/R‐induced neural injury in mice [27]. Neurological deficits were aggravated, and infarct volume increased. This study used a relatively simple score of 5 points, while mNSS we used was more comprehensive and more complicated. During the whole experiment, we made the results more reliable by maintaining the optimal living environment and controlling rats’ infection rate. Brain water content was used to measure the cerebral edema, which is usually caused by the destruction of the blood-brain barrier. In this experiment, on the 3 day after MCAO, the nicorandil treatment could reduce brain water content more than MCAO group, which is statistically significant. Research by Owjfard et al. showed the nicorandil treatment could reduce brain water content, but there is no statistical significance [10]. We speculate that it was caused by our use of nicorandil at 7.5 mg/kg, which is higher than the use of nicorandil (5 mg/kg) in their study.

Cerebral infarction is an acute blood flow disorder. Ischemia, hypoxia and energy depletion can lead to plasma membrane depolarization, oxidative stress, excitotoxicity, calcium overload and so on. Some studies have shown that excitatory neurotransmitter glutamate can activate NADPH oxidase, and reactive oxygen species (ROS) will increase and accumulate in synaptosomes [28, 29]. Therefore, even minor excitatory amino acid receptors in the presynaptic region, oxidative stress can be induced, leading to synaptic transmission disorder and neurological deficits [30]. Then the body will produce compensation, and the level of synaptic proteins would change. The interaction between proteins can complete the transmission of information, improve synaptic plasticity, and promote nerve function repair. SYP and GAP43 are proteins closely related to synaptic transmission that would change accordingly.

SYP is a transmembrane glycoprotein located on presynaptic vesicles in neurons. It participates in the formation of synaptic vesicles and stabilizes and modifies the functions of other synaptic proteins. It plays a crucial role in the formation and fusion of synaptic vesicles, and the release of neurotransmitters from vesicles [12, 31]. Previous studies have indicated that global cerebral ischemia produces a gradual decrease in SYP levels [32, 33]. Consistent with these studies’ results, cerebral ischemia decreased SYP expression in the rat hippocampus in our study, while the nicorandil significantly inhibited this change, suggesting that nicorandil exerts protective effects.

GAP43 is a vital component of axons and presynaptic terminals, where it is expressed at high levels during neuronal development and regeneration. It is one of the molecular markers of axon genesis, development and plasticity [34]. Brain damage causes the destruction of synapses, which further induces pathways that promote the plasticity of the brain to repair structures and create functional compensation. In neurons, GAP43 expression is closely related to axon growth, synaptogenesis, and nerve sprouting [35]. The expression of GAP43 increases after peripheral nerve or central nerve destruction, but the increase does not last for long periods, and GAP43 expression decreases to a basal level when compensatory synaptic connections are established [36]. Some studies have shown that GAP43 level will increase to varying degrees at 6 hours after MCAO, reaching a peak on the 7th day, and then significantly decreasing on the 21st day. We chose the 7-day time point after surgery for observation, and we detected a higher level of GAP43 in the MCAO group than in the Sham group, consistent with a previous study [37]. Additionally, the administration of nicorandil facilitated this process. However, only two synaptic proteins were tested in this study, and the evidence for this drug is not sufficient. Further investigations on other synaptic proteins and synaptic length are strongly recommended.

Ischemic stroke could lead to cognitive and motor impairments [38]. The destruction of synapses delays delivery of transmitters, which in turn leads to learning and memory deficits in rats, such as decreased spatial learning and memory performance in the MWM. In our study, rats that received nicorandil exhibited a shorter latency to find hidden platform than rats in the MCAO and MCAO+Vehicle groups, further confirming that nicorandil is effective at promoting functional recovery after cerebral infarction. This finding is consistent with a previous study showing that nicorandil activates hippocampal KATP channels and is involved in learning and memory [39]. The beneficial effects may be related to a decrease in neuronal damage, the prevention of central cholinergic dysfunction, and a decrease in oxidative damage. Nicorandil improved synaptic remodeling, which plays a vital role in the functional sensorimotor recovery after MCAO.

Studies have shown that neurogenesis in the SVZ area would increase in MCAO model. Although neurogenesis may be rare, they still produce some self-repair functions [40, 41]. Neural stem cells in the SVZ to proliferate, migrate to the peri-infarct zone, and differentiate into mature neurons. BrdU records newly proliferated cells [42]. Our results show that BrdU positive area in the SVZ is enhanced after MCAO, and nicorandil promotes it. NeuN is a recognized marker of mature neurons. We observed a decrease in the level of NeuN in the peri-infarct area on 14 days after MCAO. The results showed that MCAO could reduce NeuN level, while the application of nicorandil increased it. We did not further explore why NeuN had such a change. We speculated that this might be related to the inhibition of NeuN decrease or the promotion of neurogenesis by nicorandil. Martínez-Moreno et al. have shown that KATP opener, Diazoxide, could protect neurons by being anti-inflammatory, and it may promote neurogenesis by reducing the synthesis of inflammatory compounds [43]. Further research on the role of nicorandil in ischemic stroke needs to be explored.

The present study has some limitations. We did not investigate possible mechanisms underlying the effects of nicorandil. It was suggested to play an important role in neuroprotection after stroke by activating the PI3K/Akt signaling pathway [44, 45], which remains further explored.

## Supporting information

S1 Raw images The mRNA of NeuN, SYP and GAP43; the western blots of NeuN, SYP and GAP43. (PDF)
