# Supplementary material for: The KATP channel opener, nicorandil, ameliorates brain damage by modulating synaptogenesis after ischemic stroke
Source: PLoS One. 2021 Jan 26;16(1):e0246019. doi: 10.1371/journal.pone.0246019 (PMC7837460; doi:10.1371/journal.pone.0246019)

1： Sham 2: MCAO 3: MCAO+Nicorandil 4: MCAO+Vehicle

NeuN PCR: (Fig 3B)

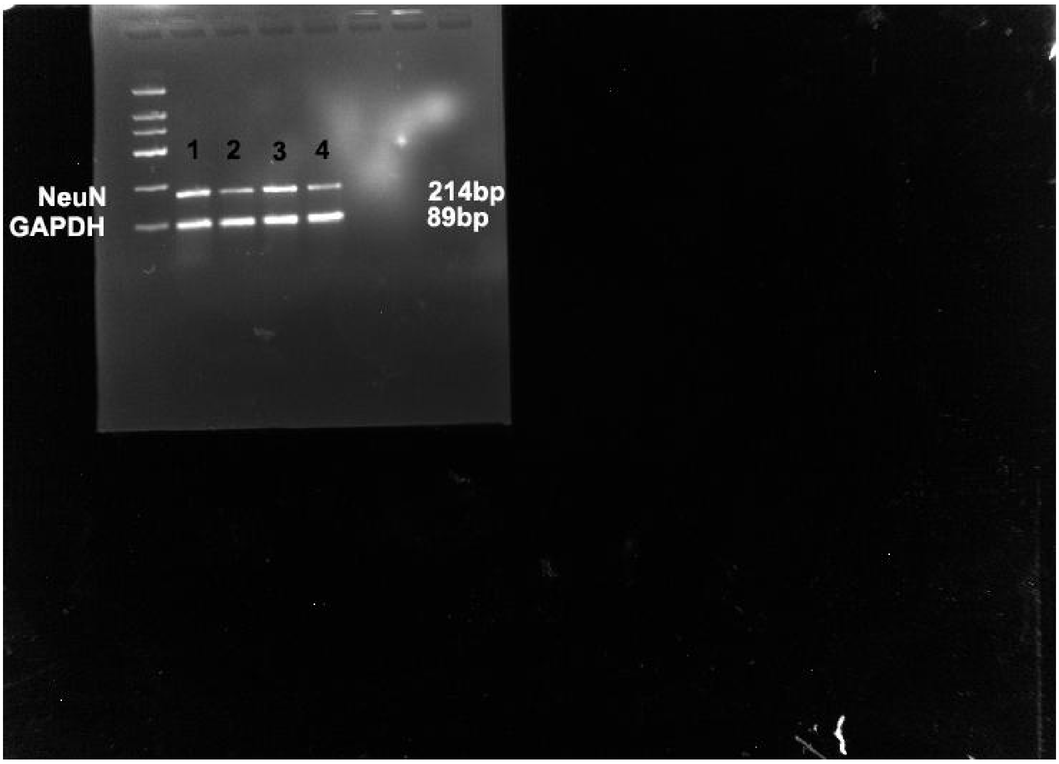

SYP PCR: (Fig4B)

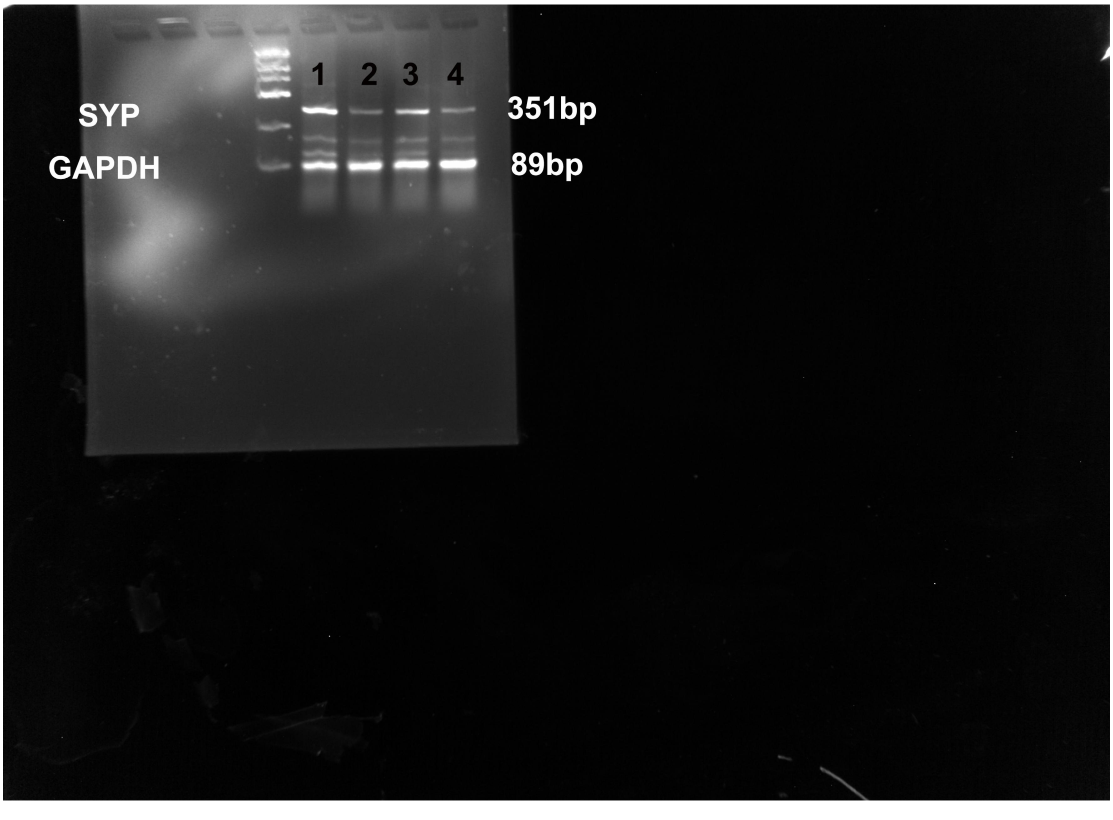

GAP43 PCR: (Fig 5B)

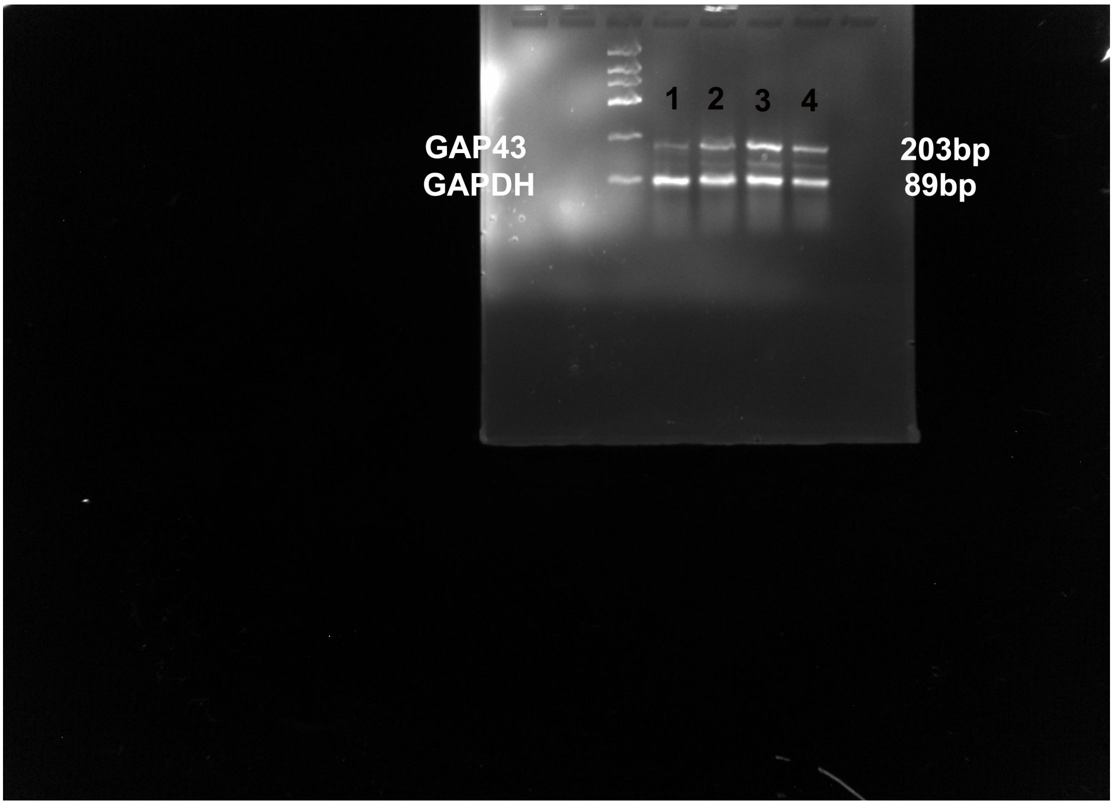

Western blot

1： Sham 2: MCAO 3: MCAO+Nicorandil 4: MCAO+Vehicle

NeuN western blot:(Fig3D)

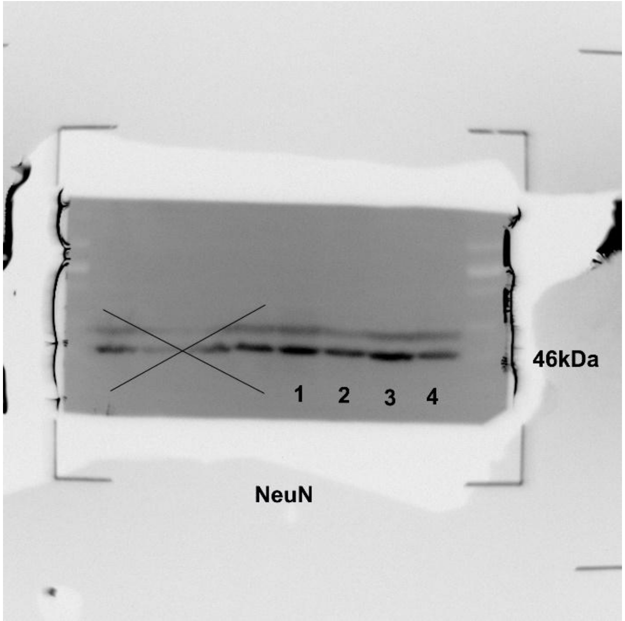

GAPDH：

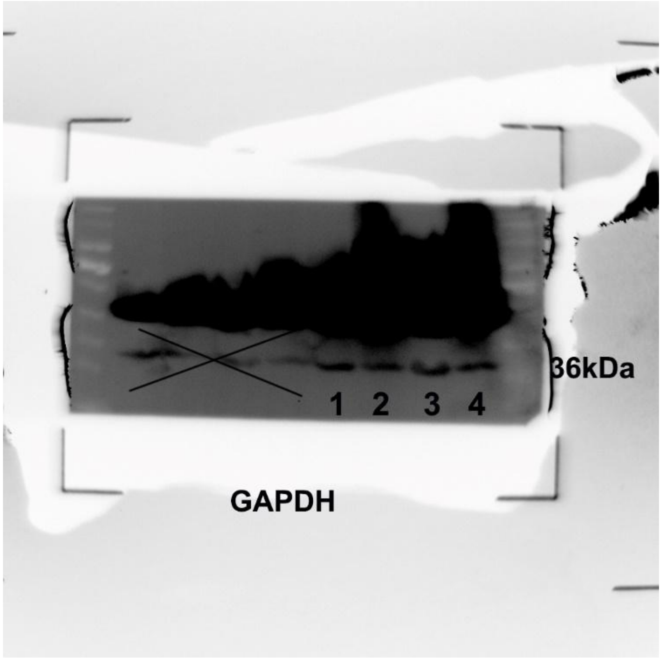

SYP western blot: (Fig4D)

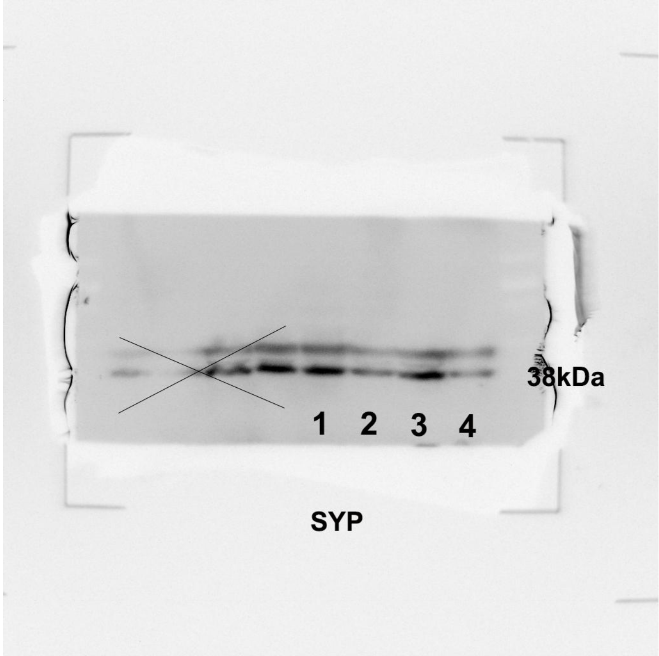

GAPDH：

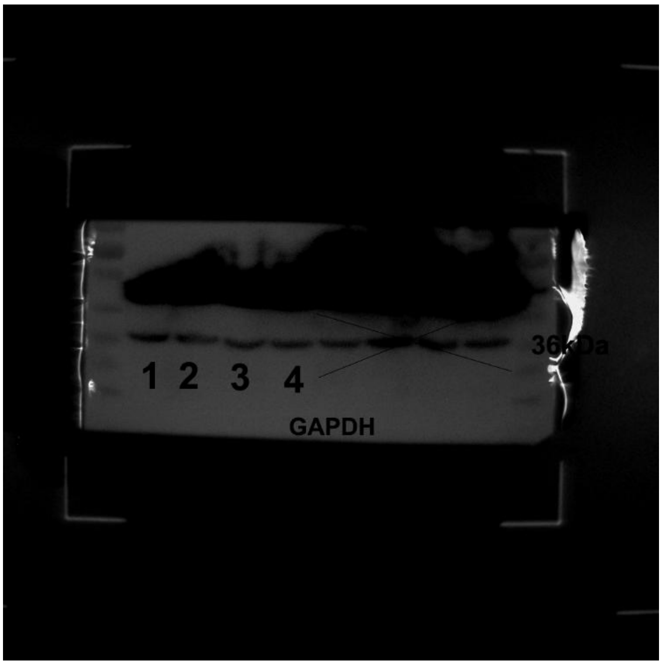

GAP43 western blot: (Fig5D)

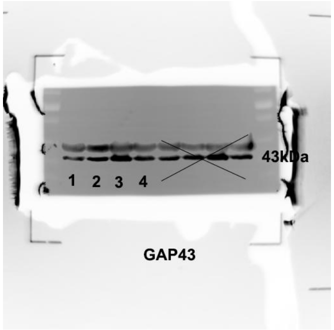

GAPDH:

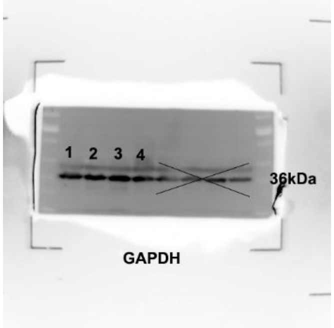

Supplement: S1 Raw images — (PDF) [file pone.0246019.s001.pdf]
